# Untranslated region engineering strategies for gene overexpression, fine-tuning, and dynamic regulation

**DOI:** 10.71150/jm.2501033

**Published:** 2025-03-28

**Authors:** Jun Ren, So Hee Oh, Dokyun Na

**Affiliations:** Department of Biomedical Engineering, Chung-Ang University, Seoul 06974, Republic of Korea

**Keywords:** UTR engineering, transcription, translation, gene expression, synthetic biology

## Abstract

Precise and tunable gene expression is crucial for various biotechnological applications, including protein overexpression, fine-tuned metabolic pathway engineering, and dynamic gene regulation. Untranslated regions (UTRs) of mRNAs have emerged as key regulatory elements that modulate transcription and translation. In this review, we explore recent advances in UTR engineering strategies for bacterial gene expression optimization. We discuss approaches for enhancing protein expression through AU-rich elements, RG4 structures, and synthetic dual UTRs, as well as ProQC systems that improve translation fidelity. Additionally, we examine strategies for fine-tuning gene expression using UTR libraries and synthetic terminators that balance metabolic flux. Finally, we highlight riboswitches and toehold switches, which enable dynamic gene regulation in response to environmental or metabolic cues. The integration of these UTR-based regulatory tools provides a versatile and modular framework for optimizing bacterial gene expression, enhancing metabolic engineering, and advancing synthetic biology applications.

## Introduction

Bacterial protein expression is a fundamental process in diverse biotechnological applications, involving the coordinated interplay of transcription, translation, and the metabolism of mRNAs and proteins (Arendt et al., 2016; Buccitelli & Selbach, 2020; Li & Borodina, 2015; Vo et al., 2019). In the industrial production of therapeutic and diagnostic recombinant proteins, genes are engineered to achieve high expression levels. However, the expression or yield of heterologous proteins is often suboptimal due to multiple factors, including promoter strength, codon usage of the host species, and other regulatory elements (Lee et al., 2021c). In metabolic engineering, enzyme genes should be optimized to ensure efficient expression, minimizing metabolic disturbances and maximizing metabolite production (Bahiri-Elitzur & Tuller, 2021; Saleski et al., 2021).Similarly, in synthetic biology, precise and dynamic gene regulation is essential for enabling programmed cellular behaviors (Xu & Qi, 2019).

Recently, untranslated regions (UTRs) have been identified as crucial regulators of gene expression, offering a promising avenue for precise expression optimization and effective genetic control (Tietze & Lale, 2021). The 5′ UTR, located upstream of a coding sequence (CDS), plays a significant role in regulating gene expression. The 5′ UTR of mRNAs contains multiple functional elements that play crucial roles in translation and, in some cases, transcription (Tietze & Lale, 2021). One of the most critical processes occurring in the 5′ UTR is translation initiation, where the translation machinery assembles at the SD sequence with the assistance of several accessory proteins (Wen et al., 2021). Various 5′ UTR elements have been identified, which enhance protein expression by facilitating transcription or translation (Le et al., 2020; Lee et al., 2024; Yang et al., 2021a), which can be engineered for fine-tuned gene expression, supporting precise expression optimization (Li et al., 2020; Rao et al., 2024; Yoo et al., 2020; Zhu et al., 2024) and which function as molecular "on" or "off" switches, responding to specific metabolites to regulate downstream gene expression (Kent & Dixon, 2019; Wang & Simmel, 2022). As a result, recent studies have focused on UTR engineering as an additional regulatory layer, complementing conventional approaches like promoter selection and codon optimization, to improve recombinant protein yield, fine-tune enzyme levels, and dynamically regulate gene expression (Liu et al., 2024; Winkler & Breaker, 2005; Yang et al., 2021a; Yoo et al., 2020).

**Fig. 1.**
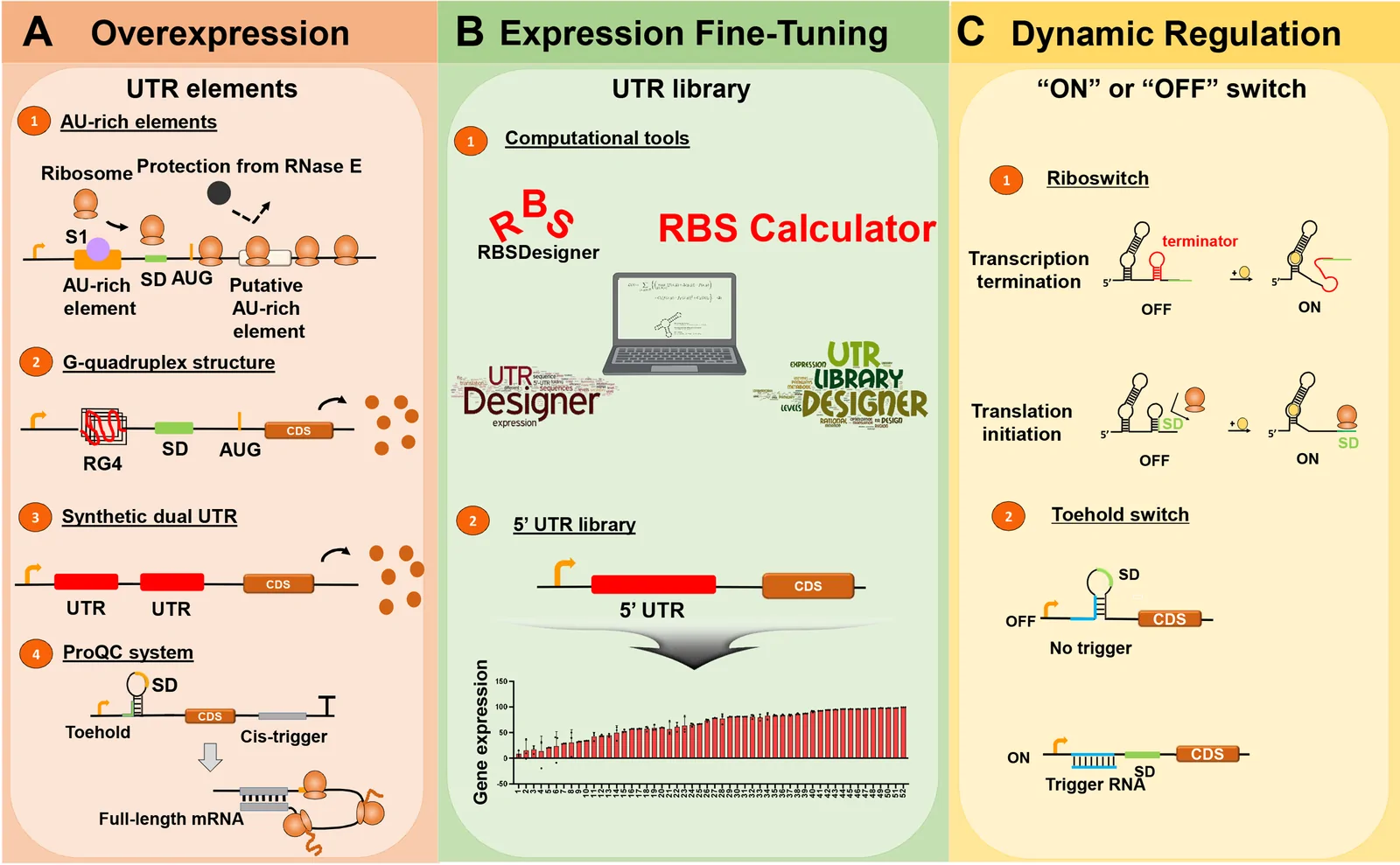
Schematic illustration on recent UTR engineering strategies. (A) Several strategies utilizing diverse UTR elements, such as AU-rich elements, RG4 structures, synthetic dual UTRs, and the ProQC system, have been employed for overexpression. (B) A 5’UTR library generated using diverse computational tools can enable fine-tuned gene expression. (C) Riboswitches and toehold switches are “on” or “off” switches that dynamically regulate gene expression.

In this review, we explore UTR engineering strategies and their underlying mechanisms across three key applications: protein overexpression, expression fine-tuning, and dynamic gene regulation (Fig. 1). Traditionally, promoters and coding sequences have been the primary targets for genetic engineering to achieve desired expression levels or regulate gene expression (Kudla et al., 2009; Lee et al., 2021c; Sung et al., 2016). However, recent discoveries have highlighted the critical roles of UTR elements in gene expression, opening new avenues for precise expression control in bacteria (Choe et al., 2022; Liu et al., 2024, 2025). Advances in genetic engineering strategies utilizing UTR modifications have provided additional layers of control over gene expression. A summary of these recent approaches is presented in Tables 1 and 2, followed by a detailed discussion below.

## Overexpression strategies

The overexpression of recombinant proteins, including therapeutics and diagnostics, is essential in the biotechnology industry. Typical approaches involve using strong promoters, such as the T7 promoter in *E. coli* BL21(DE3) strains, and optimizing the codon usage of heterologous genes to match the host strain (Du et al., 2021). However, despite these strategies, heterologous genes often fail to express efficiently or are expressed at low levels (Watts et al., 2021). To address these challenges, UTR engineering has been explored as a means to enhance protein expression.

### AU-rich elements

Secondary structures surrounding the SD region play a crucial role in translation initiation. A strong hairpin structure can sequester the SD sequence, preventing ribosome binding and reducing translation efficiency (Chelkowska-Pauszek et al., 2021). The insertion of an AU-rich element upstream of the SD sequence has been shown to enhance protein expression by promoting SD accessibility (Lee et al., 2021b). AU-rich elements have been identified in various genes, including *febB* (Hook-Barnard et al., 2007), *sodB* (Lee et al., 2021b), *malt* (Lee et al., 2021b), and *mlcR* (Kondo & Shimizu, 2023). When inserted upstream of the SD sequence, these elements significantly increase protein expression by facilitating ribosome recruitment.

Interestingly, AU-rich sequences are also known RNase E binding sites, which should theoretically lead to mRNA degradation (Baker & Mackie, 2003). However, studies have shown that AU-rich elements can enhance mRNA stability, despite their potential association with RNase E (Hook-Barnard et al., 2007; Kondo & Shimizu, 2023). One possible explanation is the interaction among ribosomal protein S1, Hfq protein, and RNase E, which share the same binding sites. Overexpression of ribosomal protein S1 has been found to suppress RNase E-dependent mRNA degradation (Delvillani et al., 2011). Additionally, ribosomes and Hfq proteins binding to AU-rich elements may shield mRNAs from degradation (Lee et al., 2021b). Another hypothesis suggests that AU-rich elements may form protective hairpin structures, though the precise mechanism remains unclear (Kondo & Shimizu, 2023). When these structures were disrupted, mRNA degradation increased (Kondo & Shimizu, 2023). Collectively, these findings suggest that AU-rich elements enhance translation through both structural effects and interactions with cellular components.

The AU-rich sequence elements, including their length and composition, however, need to be optimized for improved translation efficiency. The relatively long AU-rich element, for example, derived from the *sodB* gene may enhance RNase E accessibility to mRNA, potentially reducing mRNA stability, since AU-rich regions are also recognized by RNases for degradation. Therefore, their length as well as composition should be optimized for their practical use in protein overexpression.

### RG4 structure in 5′ UTR and Corn sequence in 3′ UTR

Unlike the conventional notion that an open SD region enhances translation, certain structured elements such as R-loop/G-quadruplex (RG4) structures can simultaneously promote both transcription and translation (Lee et al., 2020, 2024). Originally identified in eukaryotes, RG4 structures act as internal ribosome entry sites (IRES) that facilitate translation initiation independent of a transcription start site (TSS) (Al-Zeer et al., 2019; Bhattacharyya et al., 2015; Cammas et al., 2015). Although several studies have provided evidence of RG4 formation during transcription (Wanrooij et al., 2012; Zhao et al., 2017), their direct impact on transcription remains unclear.

In *E. coli*, the incorporation of a mutant RG4 structure within the 5′ UTR significantly enhanced translation in a T7-based in vitro translation system (Lee et al., 2024). Furthermore, inserting a hairpin structure upstream of RG4 structure further boosted translation by up to 12-fold, depending on the stem length and predicted folding energy (ΔG). The physical barrier model suggests that bulky structures within the 5′ UTR guide ribosome movement toward the start codon, thereby increasing translation output. This effect is attributed to structural influence rather than alterations in ribosome affinity, ribosome-binding site accessibility, or mRNA stability. In addition, Zhang et al investigated the relationship between RNA stability and 5′ UTR secondary structure by modifying the hairpin structure in 5′-UTR (Zhang et al., 2021). After evaluating varying lengths between the 5′-end hairpin and SD, the identified optimal distance was 25–30 nucleotide-long with a GC-content of 32%. With this optimal hairpin structure (named 5′ UTR stabilizer hairpin) enhanced GFP expression by up to 8-fold.

In addition to the 5′-UTR modification, incorporation of a homodimerizable RG4 RNA aptamer ("Corn") into the 3′ UTR also improved mRNA stability and reduced degradation by RNases (Sjekloća & Ferré-D’Amaré, 2019). When a five-repeated Corn sequence was added to the 3′ UTR, GFP expression increased by more than 20-fold. This enhancement was attributed to the increased mRNA half-life, which was extended by four-fold compared with non-engineered mRNA.

The correlation between a strong hairpin structure at the 5' UTR end and mRNA stability was discovered decades ago (Emory et al., 1992). RG4 also functions as a structural effector, enhancing mRNA stability and increasing expression. However, its strong helical structure complicates the incorporation of other elements like riboswitches into the 5' UTR. This is because ribonucleotides forming strong intramolecular interactions within the RG4 structure can also interact with other elements, disrupting their structures. Therefore, for the extended application of RG4, its sequences and structures should be further optimized to minimize unexpected interactions within the 5' UTR while ensuring enhanced mRNA stability.

### Synthetic dual UTR for enhancing both transcription and translation

The 5′ UTR plays a crucial role in both transcription and translation, as these processes are tightly coupled in bacteria (Proshkin et al., 2010). The initially transcribed sequence (first 15 nucleotides of the 5′ UTR) interacts with the promoter and corresponding DNA region, influencing transcription efficiency (Goldman et al., 2009; Hsu et al., 2006). Meanwhile, ribosome recruitment and mRNA stability are regulated by various elements within the 5′ UTR (Kosuri et al., 2013; Kozak, 2005).

To enhance gene expression, a library of over 400,000 randomized UTRs has been screened in *E. coli* (Le et al., 2020). In this approach, two genes (*celB* and *bla*) were expressed polycistronically—*celB* for screening high transcription efficiency and *bla* for screening high translation efficiency. After identifying optimal UTRs for transcription and translation, they were concatenated to form a synthetic dual UTR. This synthetic dual UTR significantly increased mCherry expression by up to 50-fold. When tested in *Pseudomonas putida* KT2440, a similar transcription-translation synergy was observed, although the effect was weaker due to differences in gene expression machinery.

When utilizing optimized UTRs for gene expression, several potential limitations should be considered. First, the effectiveness of a given UTR sequence may vary depending on the genetic context, including the promoter, coding sequence, and host organism. UTRs optimized in *E. coli* may not function as efficiently in other bacteria due to differences in transcription and translation machinery. Additionally, some UTRs may inadvertently introduce regulatory elements, such as riboswitches, which could interfere with gene expression in unpredictable ways. Thus, the performance of optimized UTRs may vary under different environmental conditions or growth phases. Thus, further research should focus on the optimization of the sequences to remove regulatory elements within the UTRs.

### Synthetic protein quality control system for enhanced translation

The coupled processes of transcription and translation often result in truncated proteins due to incomplete mRNA sequences, inevitably leading to the production of non-functional polypeptides and reducing the efficiency of full-length protein synthesis (Cambray et al., 2018; Dong et al., 1995; Vind et al., 1993). To address this issue, a synthetic protein quality control (ProQC) system was developed to ensure that translation occurs only when full-length mRNAs are present, thereby minimizing abortive translation caused by truncated mRNAs (Yang et al., 2021a). The ProQC system consists of two key elements: a toehold switch located in the 5′ UTR and a cis-trigger sequence in the 3′ UTR. The toehold switch forms a hairpin structure that masks the SD sequence, preventing translation initiation (see 3.3.2 *Toehold switches* for details). The cis-trigger is a complementary sequence to the toehold switch, allowing it to hybridize and subsequently release the SD sequence, enabling ribosome recruitment and translation. In the case of truncated mRNAs, the absence of the cis-trigger prevents the unfolding of the toehold switch, thereby blocking translation. Conversely, when full-length mRNA is transcribed, the cis-trigger hybridizes with the toehold switch, opening its hairpin structure and initiating translation. The implementation of the ProQC system significantly increased the proportion of intact proteins, improving overall protein levels by up to 2.5-fold.

The ProQC system was also utilized to enhance the expression of heterologous enzyme genes involved in the biosynthesis of metabolites such as 3-hydroxypropionic acid (3-HP), violacein, and lycopene in *E. coli*. Malonyl-CoA reductase, encoded by *mcr* from *Chloroflexus aurantiacus*, catalyzes the conversion of malonyl-CoA through a two-step enzymatic reaction to produce 3-HP. When *mcr* was expressed using the ProQC system, 3-HP production increased by 1.6-fold due to the enhanced synthesis of functional malonyl-CoA reductase. Similarly, the polyketide synthase *vioB* originated from *Chromobacterium violaceum*, the largest enzyme in the violacein biosynthetic pathway, which consists of five genes (*vioA*, *vioB*, *vioC*, *vioD*, and *vioE*), exhibited a 2.3-fold enhancement in the conversion of L-tryptophan to violacein when expressed using the ProQC system. For improved lycopene production, a fused enzyme consisting of phytoene synthase, encoded by *crtB* from *Lamprocystis purpurea*, and phytoene desaturase, encoded by *crtI*, was expressed using the ProQC system. Co-expression of this fusion enzyme with geranylgeranyl diphosphate synthase, encoded by *crtE*, resulted in a 2.2-fold increase in lycopene titer. These results underscore the importance of high functional enzyme production in optimizing the metabolic synthesis of valuable biomolecules and also indicate that the ProQC system enhances protein production without modifying the transcription and translation machinery, making it applicable across various genes and bacterial species.

While the ProQC system offers a promising approach to ensuring full-length protein synthesis and enhancing metabolic enzyme production, its effectiveness depends on the specific design of the toehold switch and cis-trigger sequences. Therefore, the system requires careful optimization for each target gene, necessitating additional engineering efforts. In addition, similar to RG elements, its strong helical structure complicates the incorporation of other elements, highlighting the need for further studies on its compatibility with other 5′ UTR elements.

## Expression fine-tuning strategies

From an industrial perspective, high-yield protein production is generally advantageous. However, certain proteins exhibit toxicity to the host, necessitating their expression at controlled levels to prevent cellular stress. Additionally, in synthetic metabolic pathways, enzymes must be expressed in a balanced manner to optimize reaction rates, maximize the titer of the desired products, and prevent the accumulation of toxic intermediates. To achieve precise regulation of gene expression, computational models and various UTR libraries have been developed to enable fine-tuned control over expression levels. However, UTR libraries remain a challenge requiring further refinement, as they are designed for static fine-tuning rather than dynamic optimization in response to environmental changes. Thus, there is also a need for UTR libraries incorporating other 5’ UTR elements such as riboswitches (Hockenberry et al., 2018).

### Computational models

As discussed earlier, translation is a complex process involving multiple cellular proteins. Among the various factors influencing translation efficiency, several computational models have been developed to predict gene expression levels based on the structural properties of mRNA. Notable models include RBSDesigner (Na & Lee, 2010), RBSCalculator (Salis, 2011), UTRDesigner (Seo et al., 2013), and UTR library Designer (Seo et al., 2014) (Table 3). These models mathematically assess the strength of secondary structures around the SD sequence, as well as the accessibility and affinity of ribosomes to the SD, and translate these calculations into predicted protein expression levels. As bacterial genes are grouped in an mRNA as a polycistronic manner, a computational model to predict the translation of bi- or tri-cistronic genes has been also developed (Tian & Salis, 2015). Recently, the advance in machine learning methods, translation prediction models for eukaryotes, in which larger expression-related data are available, have been developed including CRMnet, EPInformer, and ENGEP (Ding et al., 2023; Lin et al., 2024; Yang & Zhang, 2023). Thus, more accumulation of expression data in prokaryotes would allow for developing machine learning models for prokaryotes.

By utilizing these tools, researchers can design custom UTR sequences to achieve desired expression levels, as these computational tools are publicly accessible. For example, the RBS of the *ppc* gene was optimized by RBSDesigner to enhance 3-aminopropionic acid (3-AP) production in *E. coli* (Song et al., 2015). The *ppc* gene encodes phosphoenolpyruvate carboxylase, which converts phosphoenolpyruvate to oxaloacetate. Oxaloacetate is then converted to aspartate by *aspC* and subsequently to 3-aminopropionic acid (3-AP) by *panD*. This pathway allows oxaloacetate production while bypassing the TCA cycle. However, this bypass may disrupt cellular metabolism and affect cell growth. Therefore, optimizing *ppc* expression is crucial for balancing cell growth and 3-AP production. The optimized *ppc* UTR, designed by RBSDesigner, enabled both rapid cell growth and high 3-AP production.

RBSCalculator was employed to design new RBS sequences for *fabH* and *fabZ*, to enhance fatty alcohol production in *E. coli* (Chen et al., 2022b). These genes encode β-ketoacyl-ACP synthase III and monofunctional dehydratase, which are intermediate enzymes in the synthesis of fatty acyl-ACP from acetyl-CoA. Fatty acyl-ACP is then converted to fatty alcohol by the *far* gene encoding fatty acyl-ACP reductase. The overexpression of *fabH* and *fabZ* may accumulate fatty acyl-ACP that is toxic to the cell (Liu et al., 2016), and thus the expression of *fabH* and *fabZ* should be well-balanced with the level of *far* to avoid the accumulation of fatty acyl-ACP. The tailored RBS sequences of *fabH* and *fabZ* enabled to achieve optimized expression of *fabH* and *fabZ*, leading to high production titer of fatty alcohol.

In *E. coli*, the production of itaconic acid from acetate via the TCA cycle was enhanced through the over-expression of multiple genes designed using UTRDesigner (Noh et al., 2018). For itaconic acid production, the acetate assimilation pathway and glyoxylate shunt pathway were amplified by overexpressing key pathway genes (*acs*, *gltA*, and *aceA*). Unliked above examples, in the study those genes should be overexpressed to ensure efficient conversion of acetate to itaconic acid. Thus, the UTRDesigner was used to design RBS sequences for those genes ensuring high gene expression. As a result, the engineered *E. coli* produced significantly high level of itaconic acid.

Though the computational models have proven practically applicable to diverse biotechnological applications, they primarily focus on structural effects on translation and do not account for other regulatory factors influencing gene expression. Consequently, their predictive accuracy remains insufficient, necessitating experimental validation to confirm the designed UTRs' effectiveness.

### UTR libraries for gene expression diversification/optimization

***E. coli*:** *E. coli* is a widely used model organism due to its relatively fast growth, well-characterized genetic system, and the availability of established microbial engineering techniques (Adamczyk & Reed, 2017; Ren et al., 2023). These advantages make it an ideal platform for metabolic engineering and synthetic biology, and thus *E. coli* has been widely used in bioproduction, bioremediation, and theragnostics (Lynch et al., 2023; Pontrelli et al., 2018; Sharma et al., 2024)

Promoters are commonly used to fine-tune gene expression, as they are less affected by downstream coding sequences. To date, various mutant and synthetic promoters have been developed to achieve diverse protein expression levels (Deng et al., 2021; Lee et al., 2021c; Sung et al., 2016). However, the number of available promoters is limited, and certain promoters are influenced by cellular proteins, leading to expression interference. Therefore, constructing a UTR library provides an alternative approach to achieving precise gene expression control.

In one study, a library of 41 synthetic 5′ UTRs with a wide range of theoretical translation initiation rates was designed using RBSCalculator (Chen et al., 2022a; Salis, 2011). These sequences were fused upstream of various reporter genes (*lacZ*, *txAbF*, and *msfGFP*) under an inducible promoter to standardize transcriptional input. Despite uniform transcriptional regulation, protein expression levels varied significantly, with up to a 25-fold difference between constructs carrying the same theoretical translation initiation rates. One possible explanation is that the coding sequence itself influences translation efficiency due to its interaction with the 5′ UTR. For example, N-terminal coding sequences can alter secondary structures around the SD sequence, thereby affecting translation initiation efficiency (see *3.1.1 AU-rich elements*) (Mutalik et al., 2013; Na & Lee, 2010; Salis et al., 2009).

To mitigate this issue, a tunable UTR library was constructed to minimize interference from coding sequences (Yoo et al., 2020). Since the N-terminal region of the coding sequence can affect translation efficiency, it was necessary to determine the minimal distance required between the SD sequence and the coding sequence to reduce structural interference. A computational model (RBSDesigner) estimated this minimal distance to be approximately 300 nucleotides (Na et al., 2010; Yoo et al., 2020). To validate this, leader sequences of varying lengths, containing a His-tag for protein purification, the B-domain from *Staphylococcus aureus* for solubilization, and additional dummy sequences, were fused to multiple target genes (*gfp*, *mCherry*, *tdTomato*, and a *kanamycin-resistance* genes). When the leader sequence exceeded 288 nucleotides, protein expression levels were no longer influenced by the downstream coding sequence. Using this optimized leader sequence, diverse 5′ UTR sequences were designed the computational tool (Na & Lee, 2010), and their expression levels were verified using GFP. The constructed tunable gene expression cassettes incorporated eight different UTRs and three different promoters, resulting in 24 expression constructs capable of achieving relative expression levels ranging from 0.001 to 1, independent of the downstream coding sequence.

The applicability of these tunable expression cassettes was demonstrated in optimizing the expression of key metabolic enzymes in biosynthetic pathways. Specifically, the optimal expression levels of lysine decarboxylase for cadaverine biosynthesis and pyrroline-5-carboxylate reductase for *L*-proline biosynthesis were identified. Using the optimized expression cassettes, cadaverine and *L*-proline titers increased by 72% and 28% when compared with the previous highest titer, respectively.

Recently, 5′ UTR libraries were generated using UTR Library Designer to fine-tune the expression of the TetR-family repressors PhlF and McbR (Lee et al., 2021a). These repressors were used to control metabolic flux, enhancing the production of lycopene (a natural carotenoid colorant) and 3-hydroxypropionic acid (a precursor for industrial production of various chemicals) in *E. coli*. PhlF represses *gapA* (glyceraldehyde-3-phosphate dehydrogenase) and the *fabHDG* operon, which includes *fabD* (malonyl-CoA-acyl carrier protein transacylase), *fabH* (β-ketoacyl synthase III), and *fabG* (3-oxoacyl-reductase). Meanwhile, McbR represses *gltA* (citrate synthase) by targeting the *gltA* operon. Regulating growth-essential metabolism, such as energy and cofactor generation, requires precise flux control rather than simple overexpression or deletion strategies. To achieve this, the genomic *gapA* gene, which converts glyceraldehyde-3-phosphate into pyruvate, was precisely controlled using varying expression levels of the PhlF repressor. Since lycopene production begins with the condensation of equimolar pyruvate and glyceraldehyde-3-phosphate and the consumption of those metabolites reduces cell growth. The optimal PhlF expression, designed using the UTR Library Designer, resulted in a 2.82-fold increase in lycopene production compared with the parental strain. 3-HP is synthesized from acetyl-CoA and malonyl-CoA, which are linked to the TCA cycle and fatty acid synthesis, respectively. The *gltA* catalyzes the condensation of the acetyl group with oxaloacetate, while the *fabHDG* is responsible for the conversion and elongation of malonyl-CoA into fatty acids. The transcription of genomic *gltA* and *fabHDG* was repressed to varying degrees by integrating *mcbR* and *phlF* repressor libraries. A strain co-expressing optimally designed *phlF* and *mcbR* with UTR modifications exhibited a 16.5-fold increase in 3-HP production compared with the parental strain.

In a study, the biosynthetic pathway of lycopene was optimized for production in *E. coli* (Yang et al., 2021b). To enhance metabolic flux toward lycopene and minimize the accumulation of toxic intermediates, key genes (*crtE*, *crtB*, and *crtI*) were optimized using a 5′ UTR library designed with UTR Library Designer (Seo et al., 2014). The highest lycopene titer of 23.9 mg/L was achieved with the expression of each gene using its optimally designed UTR sequence. In another study, efficient production of naringenin from acetate was achieved by optimizing the expression of *pckA* (phosphoenolpyruvate carboxykinase) converting oxaloacetate to phosphoenolpyruvate (Kim et al., 2024). Naringenin can be synthesized from acetyl-CoA, which is derived from pyruvate. Pyruvate is generated from phosphoenolpyruvate, which is in turn converted from oxaloacetate by phosphoenolpyruvate carboxykinase. Its excessive production can lead to extensive oxaloacetate depletion, causing metabolic imbalance between cell growth and naringenin production. To precisely fine-tune *pckA* expression, 5′ UTR variants were designed using UTR Library Designer and the best UTR sequence leading to high titer of naringenin was identified (Seo et al., 2014). The resulting strain exhibited a 49.8-fold increase in naringenin production compared with the nonoptimized strain.

***Bacillus*:** Species within the *Bacillus* genus are widely used in industrial biotechnology due to their high growth rates, robust protein secretion capabilities, and minimal fermentation medium requirements (66). Additionally, *Bacillus subtilis* and *B. licheniformis* have Generally Recognized as Safe (GRAS) status, making them ideal candidates for large-scale bioproduction. Since ribosome binding sites (RBS) directly influence translational efficiency, constructing an RBS library, as has been done in *E. coli*, is a valuable approach for fine-tuning gene expression. However, the development of robust gene expression tools in *Bacillus* species has been hindered by their unique translational mechanisms, which differ significantly from those of *E. coli* (Rao et al., 2024; Rocha et al., 1999).

To address this challenge, a synthetic hairpin RBS (shRBS) library was developed for *B. licheniformis*, enabling a dynamic expression range spanning 10,000-fold (Rao et al., 2024). The shRBS forms a hairpin structure with an SD sequence in its loop region, permanently exposing the SD sequence for ribosome binding. This structural feature not only facilitates ribosome recruitment but also protects mRNA from degradation by 5′ exonucleases, enhancing mRNA stability. By varying the folding energy of shRBS structures within a range of -7.1 to -1.4 kcal/mol, a 17.4-fold increase in expression was achieved (Xiao et al., 2020). In a subsequent study, spacer length between the SD sequence and the start codon was diversified from 4 to 18 nucleotides to further expand the shRBS range (Rao et al., 2024).

Accompanying this library, a thermodynamic model was developed to predict translation rates based on key parameters such as Gibbs free energy changes in the SD sequence, start codon identity, and spacer length (Na et al., 2010). Experimental validation in multiple *Bacillus* species, including *B. subtilis*, *B. thuringiensis*, and *B. amyloliquefaciens*, demonstrated a strong correlation (R^2^ > 0.8) between predicted and observed expression levels.

The versatility of the shRBS library was further demonstrated in enzyme production and metabolic pathway optimization (Rao et al., 2024). Expression tuning of nattokinase, alkaline protease, and enzymes involved in the pulcherriminic acid biosynthetic pathway highlighted its potential for precise metabolic flux regulation. For instance, fine-tuning *leuS* expression using shRBS constructs led to a two-fold increase in pulcherriminic acid production, when U12-9 shRBS was utilized to *leuS* gene expression.

In another study, the *B. subtilis* genome was fragmented into 30–300 bp sequences using an ultrasonic disintegrator to construct a *B. subtilis* UTR library (Liu et al., 2025). The resulting fragments were inserted upstream of the *gfp* gene and expressed in *B. subtilis*. Among the tested fragments, the one with the highest GFP intensity, which exhibited a 2.5-fold increase compared with the control UTR, contained a 128-bp sequence located upstream of the *dppA* gene. Progressive truncation experiments demonstrated that a 19-nt-long 5′ UTR was sufficient to achieve this enhancement.

To construct a *B. subtilis* UTR library, a random nucleotide substitution was introduced at every third nucleotide position within the 19-nt UTR sequence. The resulting mutant UTR library displayed expression levels that varied up to 180-fold. To evaluate its functional utility, the native UTR sequences of the genes in the *rib* operon were replaced with sequences from the library, and the performance of the synthetic *rib* operon was assessed based on riboflavin titer. Among 120 randomly selected synthetic *rib* operons, the highest riboflavin-producing variant exhibited a 4.7-fold increase in titer compared to the operon containing its original UTR sequences.

**Other species :** *Corynebacterium glutamicum* is widely used in industrial biotechnology for the production of various valuable compounds, including amino acids (Yu et al., 2021), hydroxybenzoic acids (Kallscheuer & Marienhagen, 2018), ethanol (Inui et al., 2004), and putrescine (Schneider & Wendisch, 2010). Its ability to grow on inexpensive feedstocks and its lack of endotoxins make it an attractive host for large-scale production (Matsuda et al., 2014; Yim et al., 2014). However, for effective metabolic engineering, it is crucial to remodel metabolic flux to balance cell growth and the synthesis of target compounds by fine-tuning the expression of key genes (Yu et al., 2021). Compared with model organisms such as *E. coli* and *B. subtilis*, *C. glutamicum* has a more limited genetic toolbox, posing challenges for metabolic engineering. To address this limitation, a library of native promoter-UTR sequences was developed, as promoter-5′ UTR elements play a crucial role in regulating transcription and translation, making them essential for fine-tuning gene expression (Li et al., 2020). In this study, RNA-Seq data were used to identify 90 promoter-UTR sequences (200–500 bp) with varying transcript levels. These sequences were cloned upstream of a reporter gene encoding *mCherry* to assess their expression strength. Among the 90 tested promoter-UTRs, 17 exhibited strong and stable expression, with the highest-expressing construct (P_NCgl1676_-UTR) demonstrating more than a five-fold increase in expression compared with a commonly used control (P_sod_-UTR). The development of this native promoter-UTR library, based on high-throughput sequencing, provides an expanded genetic toolkit for *C. glutamicum*, facilitating its application in industrial biotechnology and the production of valuable compounds.

*Methanosarcina acetivorans* plays a crucial role in methane production, a renewable fuel with potential applications in mitigating climate change. To enable precise regulation of gene expression in *M. acetivorans*, a library of promoter–RBS combinations was constructed (Zhu et al., 2024). Initially, 13 wild-type promoter-RBS sequences were constructed and their expression levels were determined. To further expand medium-strength expression levels, 14 hybrid promoter-RBS combinations were also generated. Additionally, the strongest promoter-RBS was further modified to create six rationally designed high-expression 5′ UTR variants. When evaluated using β-glucuronidase as a reporter, these constructs exhibited a wide dynamic range of expression, spanning up to 140-fold. Promoter-RBS library provides a valuable genetic toolkit for metabolic engineering and physiological studies in *M. acetivorans*. By offering graded and tunable expression levels, it enables the optimization of metabolic flux and supports the exploration of the biotechnological potential of *M. acetivorans* for the production of renewable fuels under controlled conditions.

## Dynamic gene regulation strategies

The UTR libraries described above function as static libraries, maintaining gene expression at a constant level. However, in many cases, it is necessary to dynamically regulate gene expression by switching genes on and off in response to cellular or environmental conditions. Such dynamic gene regulation is particularly advantageous in metabolic engineering, as it helps reduce cellular burden by deactivating unnecessary enzyme expression and adjusting metabolic flux based on the metabolic state of the cell.

Traditionally, transcription factor-promoter pairs are employed to regulate gene expression. However, the availability of transcription factors that specifically sense desired compounds is limited, and engineering transcription factors to recognize new molecules is a complex and labor-intensive process. As an alternative to protein engineering, UTR engineering offers a simpler and more easily implementable approach due to the relatively straightforward composition of UTR sequences.

Here, we introduce two types of riboregulators within the 5′ UTR that enable dynamic modulation of gene expression: riboswitches and toehold switches. However, gene regulation using riboswitches and toehold switches still presents several challenges (Chau et al., 2020; Olenginski et al., 2024). Firstly, riboswitches and toehold switches often exhibit leaky expression in the OFF state or fail to achieve full activation in the ON state. Their complex and often strong secondary structures complicate their integration with other 5′ UTR elements, as they may interfere with one another. In addition, unintended interactions between the switches and endogenous RNAs can lead to undesirable regulatory effects.

### Riboswitches

Following the discovery of RNA elements within the 5′ UTR—now termed riboswitches—which can sense metabolites such as thiamine pyrophosphate and flavin mononucleotide and regulate genes involved in their biosynthesis or transport in *E. coli* and *B. subtilis* (Nahvi et al., 2002; Winkler et al., 2002), an extensive array of riboswitches has been identified. These riboswitches respond to a diverse range of ligands, including amino acids, vitamins, nucleotides, and ions (Bu et al., 2024; Kavita & Breaker, 2023; Mandal & Breaker, 2004; Salvail & Breaker, 2023). To date, a total of 82 riboswitches have been compiled in a dedicated database (Bu et al., 2024).

Riboswitches consist of two functional components: an aptamer domain and an expression platform. The aptamer domain specifically binds to a target metabolite, inducing a structural change in the expression platform. The expression platform, in turn, contains a transcription terminator, an SD sequence, or both (Barrick & Breaker, 2007). The structural transition in the expression platform can either form or relax the transcription terminator or expose or sequester the SD sequence, thereby modulating the expression of the downstream CDS (Mandal & Breaker, 2004). Since their discovery, riboswitches have attracted significant attention for their role in bacterial metabolic regulation and their potential applications in biotechnology.

The engineering of riboswitches typically focuses on modifying the aptamer domain to alter ligand-binding specificity or optimizing the expression platform for precise regulatory control (Rode et al., 2015). Two primary approaches are used to engineer riboswitches: directed evolution and rational design. Directed evolution employs a two-step screening strategy due to the coupled nature of the riboswitch modules. First, a high-throughput selection process is used to identify aptamer variants capable of binding a target molecule from a large pool of mutants, a technique known as Systematic Evolution of Ligands by Exponential Enrichment (SELEX) (Boussebayle et al., 2019; Hoetzel & Suess, 2022). Subsequently, the selected variants are screened using reporter genes to identify sequences that exhibit the desired regulatory response to the target ligand (Boussebayle et al., 2019). Rational design, on the other hand, utilizes structural and biochemical data to predict mutations that enhance or modify ligand specificity. For example, ligand-binding pockets can be fine-tuned to accommodate small molecules or synthetic analogs, expanding the range of detectable compounds (Ceres et al., 2013). By integrating both approaches, researchers have successfully developed riboswitches with novel ligand specificities and enhanced sensitivity, making them suitable for various applications in biotechnology and medicine (Kraus et al., 2023; Pavlova & Penchovsky, 2022; Vikram et al., 2022).

Riboswitches have been applied in metabolic engineering, environmental monitoring, and bioremediation. In metabolic engineering, they enable dynamic control over metabolic fluxes in microbial production systems. For instance, riboswitches responsive to pathway intermediates have been used to optimize the production of biofuels and pharmaceuticals by reducing toxicity and improving yield (Pang et al., 2020). One example is the engineering of lysine riboswitches, including lysine-activated (Lys-A, A263) and lysine-repressed (Lys-R, R357), which were screened through dual genetic selection based on a natural lysine riboswitch from Lactobacillus plantarum and optimized by riboswitch-based mutant (Jiang et al., 2024). These riboswitches were employed to regulate the expression of aspartate kinase III (*lysC*) and homoserine dehydrogenase (*hom*), in the lysine-producing strain *C. glutamicum* OW45. As a result, lysine production increased by 35% in strain aspartate kinase III (A263-*lysC*) and 43% in strain homoserine dehydrogenase (R357- *hom*) compared to the parental strain QW45.

In environmental science, riboswitch-based biosensors have been developed for the detection of pollutants and toxins (Thavarajah et al., 2020). These biosensors utilize engineered riboswitches to generate measurable outputs, such as fluorescence, in the presence of specific contaminants. Additionally, riboswitches have been integrated into bioremediation strategies to regulate microbial activity in response to environmental cues, enhancing the efficiency of pollutant degradation (Topp & Gallivan, 2010). In one study, an *E. coli*-based whole cell biosensor detecting hevy metals (Co^2+^/Ni^2+^) using riboswitches has been developed (Wang et al., 2021). Once the heavy metals bind to the riboswitches, the cell emits fluorescence. The heavy metal sensor can be integrated with metal-sequestering proteins for bioremediation (Thai et al., 2023; Tran et al., 2021). These systems are particularly valuable for field-deployable assays, as they are cost-effective and highly specific (Pardee et al., 2016b). These cell-based portable platforms enable on-site sensing and on-demand manufacturing, positioning riboswitches as versatile biosensors for diverse environmental applications.

### Toehold switches

A newly developed class of synthetic riboregulators, known as toehold switches, offers a highly sensitive and specific mechanism for RNA-based gene regulation (Kim et al., 2019; Walbrun et al., 2024). Toehold switches form a hairpin structure that sequesters the SD sequence and start codon, preventing ribosome binding and thereby inhibiting translation initiation. These switches are designed to detect specific RNA sequences, referred to as triggers, which are complementary to the toehold switch sequence. When the trigger RNA binds to the toehold domain, the hybridization disrupts the hairpin structure, exposing the SD sequence, thus enabling translation initiation.

An alternative variation, toehold repressors, functions oppositely by containing a strong hairpin structure upstream of an exposed RBS, which enhances translation of the downstream gene (Kim et al., 2019). Upon binding of the toehold domain with its trigger RNA, the interaction disrupts the strong hairpin stem, allowing a newly formed stable hairpin to sequester the SD sequence and repress translation. This design enables tunable gene expression regulation, expanding the utility of toehold switches in synthetic biology.

Toehold switches have been successfully applied in biosensing, particularly for the rapid and sensitive detection of coronaviruses (Park & Lee, 2021). In this approach, viral RNA is first amplified, and the toehold switch detects a specific viral sequence, which serves as a trigger. Upon binding, the switch activates the expression of β-galactosidase, resulting in a visible color change. This system, implemented on paper-based platforms using a cell-free expression system, demonstrated the ability to detect as few as 120 copies of coronavirus RNA within 70 min, including the amplification process. Similarly, toehold switches have been employed for pathogen identification and microbiome profiling, demonstrating their potential as a versatile tool for diagnostics and environmental monitoring (Hong et al., 2020; Pardee et al., 2016a; Takahashi et al., 2018).

## Conclusion

UTR engineering has emerged as a powerful strategy for optimizing gene expression at multiple levels, offering solutions for protein overexpression, expression fine-tuning, and dynamic regulation. By leveraging AU-rich elements, RG4 structures, synthetic UTR libraries, and regulatory riboswitches, it becomes possible to achieve precise control over bacterial gene expression. The integration of predictive computational tools has allowed for efficient design of synthetic UTRs to fine-tune the expression of target genes. However, there remains a significant need for large datasets to develop machine learning models, generally exhibiting better predictive power than rational models. With the accumulation of large datasets, various machine learning models incorporating not only translation initiation but also diverse regulatory factors in mRNA are expected to be developed for accurate design of bacterial UTR sequences with a desired expression level. It will also accelerate the rational design of UTR elements, enabling the development of highly efficient and adaptable expression systems. As our understanding of UTR-mediated regulation deepens, these strategies will continue to expand the capabilities of synthetic biology, driving innovations in biotechnology, industrial bioproduction, and therapeutic applications.

## Figures and Tables

**Table 1.** UTR engineering strategies in bacteria and their applications

Expression	UTR elements	Description and applications	Ref
Protein over-expression	AU-rich elements	A well-known AU-rich element is originated from *sodB* gene. S1 protein stabilizes AU-rich mRNAs by recruiting ribosomes and preventing degradation. Knockdown of S1 reduced GFP mRNA by 34% and RFP mRNA by 61%. In its absence, Hfq protein compensates to protect mRNAs from degradation.	Lee et al. (2021b)
	RG4	The enhanced translation by an RG4 structure follows a physical barrier model, where bulky structures in the 5′ UTR guide ribosome movement toward the downstream start codon, increasing translation efficiency.	Lee et al. (2024)
		Key 5′ UTR features were optimized, including a 25–30 nt spacer between the stabilizer hairpin and RBS and 32% GC content in the spacer. Additional introduction of the RG4 RNA aptamer "Corn" into the 3′ UTR, significantly enhanced protein expression. Employing an optimal 5′ UTR increased recombinant luciferase protein expression by 1.8-fold. Similarly, modifying a gene with both the optimal 5′ UTR motif and the 3′ UTR motif (5×Corn) led to a 3.4-fold increase in recombinant *Plasmodium falciparum *lactate dehydrogenase (PfLDH) protein expression.	Sjekloća & Ferre-D’ Amaré (2019)
	Synthetic dual UTRs	Two UTRs, enhancing transcription and translation, respectively, were identified from a library of <400,000 randomized UTRs in *E. coli* and concatenated. This synthetic dual UTR construct enhanced gene expression. The r31n47 dual UTR dramatically enhanced β-lactamase expression, compared with wild-type UTRs. For mCherry, transcript levels, fluorescence intensity, and half-life improved notably. Additionally, this dual UTR enhanced the solubility of both β-lactamase and mCherry proteins, further optimizing protein expression.	Lee et al. (2020)
	ProQC	The ProQC system was used to express *vioB* originated from *C. violaceum*, which converts L-tryptophan into violacein, a polyketide synthase product. This approach enhanced violacein production by 2.3-fold. Additionally, a synthetic fusion enzyme, CrtBI, was expressed using the ProQC system, while geranylgeranyl diphosphate synthase (*crtE*) was expressed, which resulted in a 2.2-fold increase in lycopene accumulation.	Yang et al. (2021b)
Expression fine-optimization	RBS	A total of 41 synthetic 5′ UTRs were designed using the RBSCalculator, with translation initiation rates rationally controlled over a 100,000-fold range, enabling precise gene expression regulation. The 5′ UTR_33k_30 exhibited the highest mRNA increasing *lacZ* by 5-fold, *txAbF* by 6-fold, and *msfGFP* by 2.5-fold.	Chen et al. (2022a)
		Optimal leader sequence was designed to minimize structure interference between SD and CDS. With the optimal leader sequence, RBS sequences were computationally designed using RBSDesigner to customize gene expression levels. Optimizing the expression levels of key metabolic enzymes encoded by *cadA* and PP3533 resulted in enhanced metabolite production. The expression-optimized strains produced 2.25 g/L of cadaverine (a 72% increase) and 2.59 g/L of L-proline (a 28% increase).	Yoo et al. (2020)
		The shRBS library achieved a 10⁴-fold dynamic range in expression strength by modifying the spacer regions between the SD sequence and the start codon and in *B. licheniformis*. To assess the shRBS library for metabolic engineering, five shRBSs were selected to fine-tune *leuS* gene expression, promoting pulcherriminic acid production in *B. licheniformis*. Additionally, the rate-limiting enzyme *YvmC* was overexpressed. The production of pulcherriminic acid varied based on the strength of different shRBSs regulating *leuS* expression, achieving a two-fold range. The highest production of pulcherriminic acid was observed when U12-9 shRBS was utilized to *leuS* gene expression.	Rao et al. (2024)
		A high-throughput screening method using 30–300 bp DNA fragments identified a novel 5′ UTR sequence from the *B. subtilis* genome, with the highest expression rate. Subsequently, a 5′ UTR library was constructed from the 5′ UTR by introducing mutations to diversify gene expression level. An artificial *rib* operon in *B. subtilisi*, in which the genes’ 5′ UTR were replaced with the high-efficient UTR. This artificial *rib* operon resulted in a 2.09-fold increase in riboflavin production. This enhancement was 4.7-fold higher compared with the operon with its original UTR sequences.	Liu et al. (2025)
		A library of 90 promoter-UTRs was constructed based on RNA-Seq data analysis. The strongest promoter-UTR sequence, P_NCgl1676-_UTR, exhibited expression levels over five times higher than P_sod_-UTR, the most commonly used strong promoter-UTR in *C. glutamicum*.	Li et al. (2020)
		A library of promoter–UTR sequences including RBS from various methanogens was constructed, which achieved a 140-fold dynamic range in expression strength in *M. acetivorans. *The strongest promoter-RBS was modified to generate six rationally designed high-expression 5′ -UTR variants. When evaluated using β-glucuronidase, they demonstrated a 140-fold range of expression strengths in *M. acetivorans*.	Zhu et al. (2024)
		5′ UTR libraries were designed using UTR Library Designer to diversify the expression levels of *phlF* and *mcbR* repressors*. *The expression levels of *phlF *and *mcbR *libraries were broadly diversified up to 18.57-fold and 15.14-fold, respectively. The most optimized strain using the libraries achieved a 2.82-fold increase in lycopene production compared with non-optimized strain. Additionally, a strain co-expressing *phlF* and *mcbR* with designed UTR sequences exhibited a 16.5-fold increase in 3-HP production compared with the parental strain.	Lee et al. (2021a)
		5′ UTR libraries of *crtE*, *crtB*, and *crtI* were generated using UTR Library Designer. For each gene, 16 different 5′ UTR sequences were designed. The highest lycopene titer was achieved by expressing *crtE*, *crtB*, and *crtI*, each using its optimally designed UTR sequence.	Yang et al. (2021b)
		To fine-tune *pckA* expression, four 5′ UTR variants were generated using UTR Library Designer. The best UTR sequence, leading to fine-tuned *pckA* expression, resulted in a strain that exhibited a 49.8-fold increase in naringenin production compared with the non-optimized strain.	Kim et al. (2024)
Dynamic expression regulation	Riboswitch	Riboswitch is a regulatory element in 5’ UTR, controlling the expression of downstream coding sequence due to its structural change upon interaction with its ligand.	Jiang et al. (2024)
		Two lysine riboswitches were utilized to activate and repress the expression of aspartate kinase III and homoserine dehydrogenase, respectively, in the lysine-producing strain *C. glutamicum* OW45. As a result, lysine production increased by 35% in strain QW48 (A263-*lysC*) and 43% in strain QW54 (R357-*hom*), compared with the parental strain QW45.	
	Toehold switches	Toehold switches are designed to detect specific RNA sequences, known as "triggers," with high sensitivity and specificity. Toehold switch was designed to detect coronavirus RNA, which acts like a trigger. The detection system implemented on a paper-cell free expression system could detect coronavirus.	Park & Lee (2021)

**Table 2.** Advantages and limitations of UTR elements in gene expression modulation

Expression	UTR elements	Advantages	Limitations	Ref
Protein Over-expression	AU-rich	Incorporating AU-rich elements into the 5′ UTR region can stabilize mRNA through S1 and Hfq proteins, thereby enhancing protein production.	The relatively long AU-rich element derived from the *sodB* gene, for example, may enhance RNase E accessibility, potentially reducing mRNA stability.	Lee et al. (2021b)
	RG4	RG4 structures act as internal ribosome entry sites, allowing translation initiation independent of a TSS. Furthermore, their incorporation into the 3′ UTR enhances mRNA stability and protects against RNase-mediated degradation.	The strong helical structure of RG4 complicates the incorporation of other elements like riboswitches into the 5' UTR, because ribonucleotides forming strong intramolecular interactions within the RG4 structure can also interact with other elements, disrupting their structures.	Lee et al. (2024)
	Synthetic dual UTRs	Dual UTRs elements enhance both transcription and translation with an optimal distance between two UTRs within the 5′ UTR region.	The concatenated UTRs may contain regulatory elements and thus the performance of optimized UTRs may vary under different environmental conditions or growth phases.	Lee et al. (2020)
	ProQC	The ProQC system ensures translation occurs only in the presence of full-length mRNAs by facilitating circularization through a toehold switch in the 5′ UTR and a cis-trigger sequence in the 3′ UTR. This mechanism enhances full-length translation, thereby improving protein quality in bacteria.	The incorporation of a toehold switch, forming a strong secondary structure, complicates its integration with other 5′ UTR elements. In addition, to further enhance full-length protein production, improving the circularization efficiency and stability of circular mRNA requires further investigation.	Yang et al. (2021b)
Expression fine-optimization	RBS	A UTR library offers a diverse set of UTR sequences, including RBSs, that can be incorporated into the 5′ UTR region to fine-tune target gene expression by varying ribosome binding affinity.	UTR libraries are designed for static fine-tuning rather than dynamic regulation.	Hockenberry et al. (2018)
Dynamic expression regulation	Riboswitch	Riboswitches regulate gene expression by binding specific metabolites, switching expression "on" or "off," and controlling downstream gene activity.	Riboswitches may exhibit leaky expression in the OFF state or fail to achieve full activation in the ON state.	Olenginski et al. (2024)
	Toehold switches	Toehold switches regulate gene expression by binding trigger RNAs, toggling expression "on" or "off," and controlling downstream gene activity.	The strong secondary structure of toehold switch complicates its integration with other 5′ UTR elements, and like riboswitches toehold switches often exhibit leaky expression in the OFF state or fail to achieve full activation in the ON state. Unintended interactions with endogenous RNAs can also lead to undesirable regulatory effects.	Chau et al. (2020)

**Table 3.** Computational tools for predicting and designing UTRs in bacterial gene expression

Software	Description	Ref	Exemplar applications
RBSDesinger	RBSDesigner is a mathematical model accounting for the thermodynamic RBS folding (30 nt from SD) and the interaction of RBS with ribosomes. The thermodynamic parameters in the model were calculated using UNAFold software package. The model’s prediction performance was R^2^=0.77 – 0.87.	Na & Lee (2010)	The RBS of the *ppc* gene, optimized by RBSDesigner, enhanced both rapid cell growth and high 3-AP production in *E. coli* by facilitating the conversion of phosphoenolpyruvate to oxaloacetate, which is then converted to aspartate and ultimately to 3-AP (Song et al., 2015).
	URL: http://ssbio.cau.ac.kr/web/?page_id=195		
RBSCalculator / Operon Calculator	RBSCalculator is a thermodynamic model composed of five energy parameters calculated from -35 to +35 nt of TIR by using NuPACK suite. Its prediction performance was R^2^=0.51 – 0.95 depending on the origin of UTR.	Salis (2011), Tian & Salis (2015)	Optimized expression of *fabH* and *fabZ* using RBS sequences designed by RBSCalculator enhanced the synthesis of fatty acyl-ACP from acetyl-CoA. Fatty acyl-ACP was then converted to fatty alcohol by the *far* gene, resulting in a high production titer of fatty alcohol (Chen et al., 2022b).
	Operon Calculator is a biophysical model based on the model of RBSCalculator to predict the translation of bi- and tri-cistronic genes. Its performance is Pearson R^2^=0.57 – 0.91.		
	URL: https://salislab.net/software/predict_rbs_calculator,		
	http://salislab.net/software		
UTRDesigner / UTR Library Designer	UTRDesigner is a thermodynamic model that accounts for several parameters, calculated from -10 to +35 nt of the TIR using NuPACK. Its performance is R² = 0.81. UTR Library Designer is a tool used to generate UTR sequences with diverse expression levels, utilizing UTRDesigner.	Seo et al. (2013, 2014)	For enhanced itaconic acid production, multiple genes (*acs, gltA,* and *aceA*) involved in acetate assimilation and the glyoxylate shunt pathway were overexpressed using optimized RBS sequences designed by UTRDesigner (Noh et al., 2018).
	URL: https://sbi.postech.ac.kr/utr_designer/, https://sbi.postech.ac.kr/utr_library/		
